# Possible role of ribosome biogenesis in the recovery from transient hepatic damage caused by ethylene glycol in rats

**DOI:** 10.1007/s11419-026-00757-4

**Published:** 2026-02-03

**Authors:** Kana Unuma, Toshihiko Aki, Nana Kobayashi, Shintaro Isa, Akihiro Tojo

**Affiliations:** 1 Department of Forensic Medicine Graduate School of Medical and Dental Sciences, Institute of Science Tokyo (ST), 1-5-45 Yushima, Bunkyo-Ku, Tokyo, 113-8519 Japan; 2 Department of Nephrology & Hypertension, Dokkyo Medical University, 880 kitakobayashi, mibu, Tochigi, Japan

**Keywords:** Ethylene glycol, Liver, Nucleolus, Ribosome, *SLC7A11*, *Gpx4*

## Abstract

**Purpose:**

Ethylene glycol (EG), a widely used industrial compound, has been implicated in accidental poisoning and homicide. Although the nephrotoxic mechanisms of EG are well characterized, its acute hepatotoxic potential remains underexplored. This study investigated histopathological and molecular alterations in the liver of rats following acute administration of EG. Possible dysfunction of an anti-oxidative pathway involving ferroptosis (glutathione peroxidase 4, Gpx4; solute carrier family 7 member 11, SLC7A11) is also examined.

**Methods:**

Male rats (8-week-old, male) were orally administered EG (8 g/kg) and euthanized at 2 and 5 days post-exposure by an administration of an overdose of anesthetic (40 mg/kg sodium pentobarbital). Serological analysis was performed to assess liver function. Liver tissues were evaluated by histology, transmission electron microscopy, and transcriptome analysis.

**Results:**

Serological findings indicated transient liver damage at 2 days post-exposure, followed by recovery at 5 days. Transmission electron microscopy revealed glycogen accumulation, corroborated by periodic acid–Schiff and periodic acid-methenamine silver staining. Histological analysis revealed an increased number of nucleoli, correlating with upregulation of ribosomal genes on microarray analysis, particularly at 5 days post-exposure. In addition, significant decreases in Gpx4 and SLC7A11 expression were observed in rat livers treated with EG and Huh-7 human hepatoma cells, suggesting reduced cellular antioxidative capacity as a contributing factor to transient liver damage.

**Conclusions:**

These findings reveal a pathway underlying EG-induced transient liver damage and suggest a possible mechanism of recovery, thereby providing new insights into EG poisoning.

**Supplementary Information:**

The online version contains supplementary material available at 10.1007/s11419-026-00757-4.

## Introduction

Ethylene glycol (EG) is a widely used industrial solvent found in antifreeze, coolants, de-icing agents, and household products, such as cold packs and paints [1]. While generally safe when used as intended, EG possesses high toxicity and has occasionally been implicated in both accidental and intentional poisoning [2–5]. Once ingested, EG is rapidly absorbed from the gastrointestinal tract, reaching peak blood concentrations within 2–6 h [6]. Acute toxicity of EG is generally consisted of three stages; manifestations of 1) CNS depression such as confusion and somnolence after several hours, 2) cardiopulmonary disfunctions such as heart failure after half-to-one day, and 3) renal failures after several days from EG ingestion [3, 7]. Approximately 80% of ingested EG is metabolized in the liver by alcohol dehydrogenase (ADH) and aldehyde dehydrogenase (ALDH), producing toxic metabolites, such as glycolaldehyde, glycolic acid (GA), and glyoxylic acid [8, 9]. These metabolites, rather than the parent compound, are the principal mediators of toxicity. In particular, GA and oxalic acid contribute to severe metabolic acidosis and calcium oxalate (CaOx) crystal deposition in tissues, most notably the kidney [10, 11].

Although nephrotoxicity is the most recognized outcome of EG poisoning, with CaOx crystals causing acute tubular necrosis and often acute kidney injury (AKI), EG toxicity is not confined to the kidney. CaOx crystals may also deposit in the liver, heart, lungs, central nervous system, and gastrointestinal mucosa [12, 13]. For example, liver degeneration, such as swollen mitochondria and centrilobular necrosis, has been reported in an animal model of EG poisoning [14, 15]. Nevertheless, both human and animal studies have demonstrated that renal CaOx crystals are not always detected—even in lethal cases or in those with high urinary GA concentrations [16]. This discrepancy highlights the need for a broader understanding of EG-induced tissue injury beyond crystal deposition alone.

Despite the established nephrotoxic mechanisms, the hepatic effects of EG remain underexplored, even though the liver is its principal site of metabolism. Moreover, the molecular responses of hepatocytes to EG metabolites—including alterations in metabolic enzymes and solute transporter expression—are poorly characterized. Therefore, this study aimed to investigate the acute hepatic histopathological and transcriptomic alterations following EG exposure in a rat model, with findings further validated in human hepatoma (Huh-7) cells.

## Materials and methods

### Animals and EG administration

All animal experiments were approved by the Institutional Animal Care and Use Committee (IACUC) of Institute of Science Tokyo (ST) (approval No. A2023-080C) and conducted in accordance with the Animal Research: Reporting of In Vivo Experiments (ARRIVE) guidelines [17, 18]. Eight-week-old male Wistar rats (220–250 g; Oriental Yeast Co. Ltd., Tokyo, Japan) were housed under standardized conditions (12-h light/dark cycle, 25 °C) with free access to food and water. The rats were randomly assigned to either the control or the EG-treated group. Each animal received a single oral dose of either 0.9% NaCl (control) or 8 or 16 g/kg EG (Fujifilm Wako Pure Chemical Corporation, Osaka, Japan). Samples were collected at 2 or 5 days after administration of EG (EG2 or EG5, respectively), after euthanasia with an overdose of sodium pentobarbital (40 mg/kg). Each group consisted of 6 male rats, with 3–5 animals per group used for each specific analysis. The 8 g/kg EG dose was selected based on a previous study investigating acute EG toxicity [19].

### Cell culture and EG administration

The Huh-7 human hepatoma cell line (RCB1366, RIKEN Bioresources Center, Tsukuba, Japan) was used as an in vitro model of EG-induced hepatotoxicity. Cells were maintained in Dulbecco’s Modified Eagle Medium supplemented with 10% fetal bovine serum, 100 μg/mL streptomycin, and 100 U/mL penicillin. EG concentrations (0.3, 0.6, and 1.2 M) were selected based on a previous report [20], in which the IC50 of EG on the viability of KB (HeLa derivative) cells was estimated at 0.45 M. Cell viability was measured using the Cell Counting Kit-8 (Dojindo, Kumamoto, Japan).

### Blood analysis

Blood samples (anticoagulant: 1.5 mg/mL K_3_-EDTA) were collected from the rats at 2 and 5 days after EG administration (8 g/kg). Plasma levels of albumin (ALB), aspartate aminotransferase (AST), alanine aminotransferase (ALT), alkaline phosphatase (ALP), lactate dehydrogenase (LDH), and γ-glutamyl transpeptidase (γ-GTP) were measured according to the standard clinical chemistry methods (Oriental Yeast Co., Ltd., Tokyo, Japan). Blood samples collected prior to organ harvesting on days 2 and 5 were immediately analyzed for pH, bicarbonate (HCO₃⁻), and anion gap (AG) using the i-STAT®1 analyzer with EC8 + cartridges (Abbott Point of Care Inc., Princeton, NJ, USA).

### Histological analysis, immunohistochemistry, and electron microscopy

Rat liver tissues were fixed in 4% paraformaldehyde, embedded in paraffin, and sliced into 2.5 µm sections. For histological analysis, the sections were stained with hematoxylin and eosin (H&E). Transmission electron microscopy (TEM) of rat liver was performed according to previously described methods [21, 22]. Briefly, approximately 1 mm^3^ of liver tissue was fixed in 2.5% glutaraldehyde, post-fixed in 1% osmium tetroxide, and embedded in epoxy resin (EPON). Ultrathin Sects. (80 nm) were stained with uranyl acetate and lead citrate and examined using a transmission electron microscope (HT7800, Hitachi High-Tech Co., Tokyo, Japan). Semi-thin Sects. (0.5 μm) of EPON-embedded tissue, prepared prior to ultrathin sectioning for TEM, were stained with toluidine blue and observed under both bright-field and dark-field microscopy. To visualize glycogen, periodic acid–Schiff (PAS) and periodic acid-methenamine silver (PAM) staining were performed after removing epoxy resin from EPON-embedded sections using a saturated KOH solution. Oil Red O staining of frozen sections was also conducted to visualize lipid droplets.

### DNA microarray and qPCR analysis

Total RNA from the livers of rats treated with or without EG was subjected to Clariom S array analysis (Thermo Fisher Scientific, Waltham, MA). Prior to analysis, RNA was purified using RNeasy Mini Kit (QIAGEN, Hilden, Germany), and RNA integrity was verified using a Bioanalyzer (Agilent, Santa Clara, CA). Transcriptome Analysis Console software (Thermo Fisher Scientific) was used for pathway enrichment analysis, and Metascape (https://metascape.org/gp/index.html#/main/step1) [23] was used for gene ontology (GO) enrichment analysis. Relative mRNA levels were measured using reverse transcription-quantitative PCR (RT-qPCR). Briefly, total RNA extracted from rat liver or Huh-7 cells using TRIzol reagent (Thermo Fisher Scientific) was used for cDNA synthesis. SuperScript II reverse transcriptase (Thermo Fisher Scientific) and oligo(dT)_15_ primers were used to synthesize cDNA from total RNA. Gene-specific primers (Table S1), SYBR Green dye (Promega, Madison, WI), and the StepOne real-time PCR system (Thermo Fisher Scientific) were used for qPCR. Relative mRNA abundances were calculated using the comparative Ct method [24].

### Immunoblot analysis

Rat liver tissues were immersed in lysis buffer [10 mM Tris–HCl (pH 8.0), 320 mM sucrose, 1 mM EDTA, 50 mM NaF, 2 mM Na_3_VO_4_, and protease inhibitor cocktail (Complete; Roche, Mannheim, Germany)] together with magnetic beads and disrupted using a bead crusher [3000 revolutions per minutes (rpm) for 10 s at room temperature, 3 cycles] (Multi-beads Shocker MB3000, Yasui Kikai, Osaka, Japan). The homogenates were centrifuged at 20,000 × g for 15 min to remove debris. Laemmli’s buffer was added to the supernatant [final composition is 0.06 M Tris–HCl (pH 6.8), 2% SDS, 3% 2-mercaptoethanol, 10% glycerol, and 0.005% BPB]. Huh-7 cells were collected in the Laemmli’s buffer, and lysates were obtained via sonication (Output, 40 kHz; Pulse Setting, continuous; Duration, 10 s; Temperature, room temperature) (SFX150HH, Branson, Brookfield, CT). The tissue as well as cell lysates were subjected to SDS-PAGE (15% gels for Gpx4, SLC7A11, GAPDH, phospho-eIFα, and eIFα; 10% gels for 4-hydroxy-2-nonenal) by the method of Laemmli [25], and transferred on to a PVDF membrane (Immobilon-P, Millipore, Billerica, MA) by the method of Towbin et al*.* [26]. Just before the use, PVDF membranes were activated through immersion in methanol for at least 20 min. For the analysis of 4-hydroxy-2-nonenal, lysates were subjected to SDS-PAGE in discontinuous (4–15%) gels (Mini-PROTEAN-TGX precast gel; Bio-Rad, Hercules, CA). The blots were blocked with 5% non-fat dry milk in Tris-buffered saline containing 0.05% Tween 20 for 30 min at room temperature (RT), then incubated with appropriate antibodies (Table S2) over night at 4 °C. The membranes were subsequently incubated with horseradish peroxidase-conjugated anti-IgG antibodies (Promega) for 30 min at RT and visualized using enhanced chemiluminescence (ECL) reagents (Western Lightning Plus, Revvity, Waltham, MA). Band intensities were quantified using Image Capture version 4 (ATTO, Tokyo, Japan).

### Statistical analysis

Dunnett’s (for comparisons vs. a control) or Tukey–Kramer (for all pairwise comparisons) post hoc test was used for multiple group comparisons. Two-tailed *P* values < 0.05 were considered statistically significant. Statistical analyses were performed using GraphPad Prism, version 9.0.0 (GraphPad Software, San Diego, CA, USA).

## Results

### Possible liver damage in EG-administered rats

We applied 8 and 16 g/kg EG orally to rats to assess the maximum sublethal toxicity, since the LD50 of EG was estimated at 6.1–13 g/kg when administered orally to rats [8]. Both rats (2 out of 2, 100%) died 1 day after the administration of 16 g/kg EG. In contrast, a total of 6 out of 8 (75%) rats survived 5 days after the administration of 8 g/kg EG. All the rats administered saline (5 out of 5) survived after the experiments. Based on these findings, 8 g/kg EG was used throughout this study. To examine EG-induced liver damage in animal models, livers were dissected from rats at 2 and 5 days after administration. Although no significant changes in body weight were observed, liver weights decreased by approximately one-half at 5 days post-EG administration (Table 1). Consistent with the decline in liver weights, which indicated liver damage, metabolic acidosis was observed in EG-administered rats, evidenced by a significant decline in blood pH, a tendency toward decreased HCO_3_^−^ concentrations, and a significant elevation in serum AG (Table 1). Taken together, these findings indicate liver damage in EG-administered rats, as evidenced by organ weight loss and metabolic acidosis.

**Table 1:** Loss of liver weight and metabolic acidosis after ethylene glycol (EG) administration

	Control	EG2	EG5
Body Weight (g)	289.5 ± 12.2	265.7 ± 10.5	281.7 ± 9.6
Liver Weight (g)	12.87 ± 0.98	12.59 ± 0.70 **** ^(vs EG5)^	7.80 ± 0.22 **** ^(vs Control)^
pH	7.573 ± 0.162	6.942 ± 0.083 ** ^(vs Control)^	6.878 ± 0.183 ** ^(vs Control)^
HCO3^−^ (mmol/L)	19.93 ± 5.75	12.73 ± 3.70	10.80 ± 3.32
AG (mEq/L)	26.40 ± 6.43	44.60 ± 2.36	48.20 ± 12.47 * ^(vs Control)^
AST (IU/L)	94.4 ± 23.6	363.5 ± 257.1 * ^(vs Control)^	231.8 ± 33.6
ALT (IU/L)	26.6 ± 1.7	82.8 ± 28.8 ** ^(vs Control)^	44.8 ± 8.4 * ^(vs EG2)^
LDH (IFCC) (IU/L)	214.6 ± 96.7	2172.8 ± 1051.4 ** ^(vs Control)^	1461.5 ± 259.6 * ^(vs Control)^

After 12 h of fasting, 8-week-old male Wistar rats received a single oral dose of 8 g/kg EG or an equal volume of saline (0.9% NaCl) and were euthanized 2 or 5 days later. Whole body and liver weights 2 and 5 days after EG administration and blood sample analysis. Sample sizes were as follows: body weight and liver weight, n = 6 per group; pH, HCO3^−^, and AG, n = 3 per group; AST, ALT, and LDH, Control (n = 5), EG2 (n = 4), and EG5 (n = 4). The means ± S.D. are shown. *P < 0.05; **P < 0.01; ***P < 0.001; ****P < 0.0001. AG, anion gap

### Transient liver injuries in EG-administered rats

**Fig. 1:**
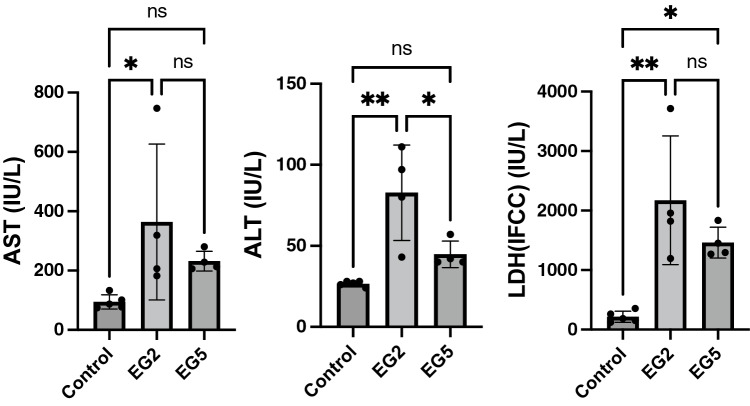
Transient liver damage after EG administration. After 12 h of fasting, 8-week-old Wistar rats received a single oral dose of 8 g/kg EG or an equal volume of saline (0.9% NaCl) and were euthanized 2 or 5 days later. Blood analysis results are shown. Each bar represents the mean ± S.D. ns, not significant; *P < 0.05; **P < 0.01. AST, aspartate aminotransferase; ALT, alanine aminotransferase; LDH, lactate dehydrogenase

Serological markers were examined to evaluate liver injury in EG-administered rats. Significant increases in AST, ALT, and LDH were observed at 2 days after EG administration (EG2 group), followed by a significant decrease in ALT and declining trends in AST and LDH in the EG5 group compared with the EG2 group (Table 1), as shown schematically in Fig. 1. Although changes were modest, similar tendencies were also noted in ALB, ALP, and γ − GT, suggesting transient (Supplementary Fig. S1) liver injury that peaked at 2 days and recovered by 5 days after EG administration.

### Histology of EG-administered rat liver

Microscopic examination of livers from EG-administered rats was conducted to identify histological features of EG-induced hepatotoxicity (Supplementary Fig. S2).

In the kidney from the EG2 group, congestion and large vacuole-like structures were observed in the cytoplasm compared with the control group (Supplementary Fig. S2A, b and e). Although nuclear morphology showed no apparent differences (Supplementary Fig. S2A, b and e), an increased number of cells contained multiple nucleoli per nucleus (Supplementary Fig. S2B).

In the kidney from the EG5 group, lesser congestion and vacuole-like structures were observed in the cytoplasm compared with the EG5 group (Supplementary Fig. S2A, c and f). As shown in Supplementary Fig. S2, the occurrence of nuclei with multiple nucleoli was significantly increased compared with that in controls (Supplementary Fig. S2B).

### Electron microscopic analysis of EG-administered rat liver

To further examine the degenerative changes observed in EG-administered rat livers, electron microscopy was performed.

**Fig. 2:**
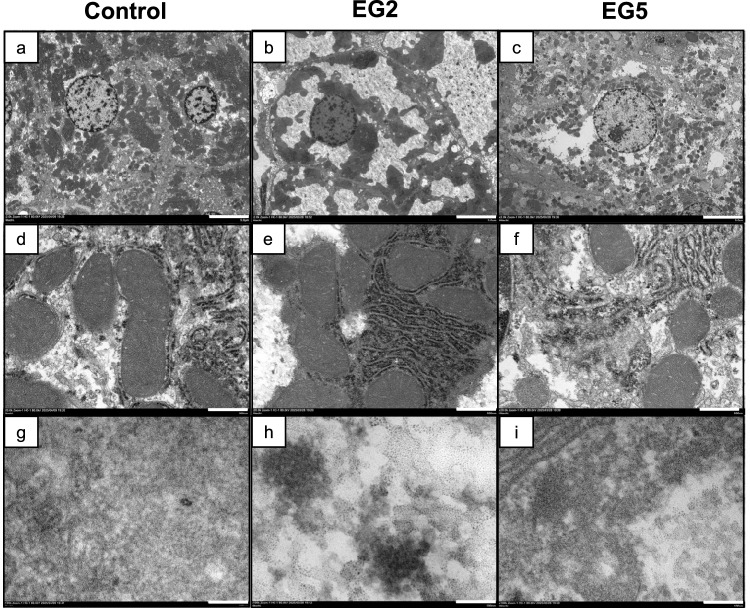
Transmission electron microscopy (TEM) of liver sections from EG-administered rats. TEM images of liver sections from control rats (control, a, d, g) and EG-treated rats 2 (EG2, b, e, h) or 5 (EG5, c, f, i) days after EG administration (8 g/kg). Bars: 5 µm (a–c), 500 nm (d–f), and 100 nm (g–i)

In the kidney from the EG2 group, the most notable finding was that hepatocyte cytoplasm contained regions of low electron density, suggestive of fluid accumulation in localized cytoplasmic areas (Fig. 2, b, e, and h). Closer inspection of these regions revealed 10–30 nm granule-like structures, consistent in size with glycogen (Fig. 2, h). Although mitochondria and ER appeared displaced to peripheral areas of the cytoplasm, no apparent mitochondrial degeneration was observed (Fig. 2, e). In contrast, the proportion of rough ER relative to total ER volume increased compared with that in controls (Fig. 2, e).

In the kidney from the EG5 group, restoration of these observations (fluid accumulation as well as increased proportion of rough ER observed in the EG2 group) to basal levels was observed (Fig. 2, c, f, and i).

### PAS, PAM, and Oil Red O staining of EG-administered rat liver

To further evaluate the low electron density region observed in the liver of the EG2 group, TEM, PAS, PAM, and Oil Red O staining were performed.

In the kidney from the EG2 group, both PAS and PAM staining showed typical glycogen staining patterns in the hepatic cytoplasm (Supplementary Fig. S3A, b and e), confirming that the granules observed by TEM (Fig. 2, h) were glycogen granules. Oil Red O staining did not stain the hepatocytes, excluding the possibility that the area contained lipids (Supplementary Fig. S3A, h). In addition, we observed crystals in the kidney from EG2 group (Supplementary Fig. S3B).

In the kidney from the EG5 group, restoration of these observations (accumulation of glycogens as well as crystals observed in the EG2 group) to basal levels was observed (Supplementary Fig. S3A, c and f).

### Bright- and dark-field microscopic observations after toluidine blue staining of EG-administered rat liver

Toluidine blue staining, which does not stain glycogen, was applied to liver sections from EG-administered rats and examined under bright-field (Supplementary Fig. S4, upper row) and dark-field (Supplementary Fig. S4, lower row) microscopy.

In the EG2 group, large areas of cytoplasm surrounding the nuclei were negative under bright-field microscopy.

In the EG5 group, the toluidine blue-negative cytoplasm showed bright birefringent granules under dark-field microscopy, suggesting the presence of oxalate (Supplementary Fig. S4, c and f).

### DNA microarray analysis of EG-administered rat liver

To further investigate the molecular mechanisms underlying transient degeneration in EG-administered rat liver, transcriptome analysis using DNA microarray was performed on liver samples from rats with and without EG administration. The top 20 most upregulated genes in the EG2 and EG5 groups are listed in Tables 2 and 3, respectively.

**Table 2:** Top 20 upregulated genes 2 days after ethylene glycol administration

No	Fold Change	Gene symbol	Description
1	29.89	*Slc3a1*	solute carrier family 3 (amino acid transporter heavy chain), member 1
2	17.19	*Adh6*	alcohol dehydrogenase 6 (class V)
3	16.43	*LOC361914*	similar to solute carrier family 7 (cationic amino acid transporter, y + system), member 12
4	11.41	*Kap*	kidney androgen regulated protein
5	10.84	*Slc7a13*	solute carrier family 7 (anionic amino acid transporter), member 13
6	9.58	*Gpx3*	glutathione peroxidase 3
7	9.5	*RGD1307603*	similar to hypothetical protein MGC37914
8	8.16	*Slc7a12*	solute carrier family 7 (cationic amino acid transporter, y + system), member 12
9	8.09	*Prg4*	proteoglycan 4
10	7.94	*Sult2a1*	sulfotransferase family 2 A, dehydroepiandrosterone (DHEA)-preferring, member 1
11	7.76	*Havcr1*	hepatitis A virus cellular receptor 1
12	7.53	*Ggt1*	gamma-glutamyltransferase 1
13	7.31	*Cyp17a1*	cytochrome P450, family 17, subfamily a, polypeptide 1
14	7.22	*LOC100911413*	deleted in malignant brain tumors 1 protein-like
15	7.17	*Mep1a*	meprin 1 alphaP
16	6.81	*Slc34a1*	solute carrier family 34 (type II sodium/phosphate cotransporter), member 1
17	6.26	*Wfdc21*	WAP four-disulfide core domain 21
18	6.13	*Cd109*	CD109 molecule
19	6.04	*Olr1662*	olfactory receptor 1662
20	5.9	*Cep112*	centrosomal protein 112 kDa

**Table 3:** Top 20 upregulated genes 5 days after ethylene glycol administration

No	Fold Change	Gene symbol	Description
1	82.88	*Akr1b7*	aldo–keto reductase family 1, member B7
2	27.13	*Sult2a6*	sulfotransferase family 2 A, dehydroepiandrosterone (DHEA)-preferring, member 6
3	19.43	*Sult2a1*	sulfotransferase family 2 A, dehydroepiandrosterone (DHEA)-preferring, member 1
4	12.98	*RGD1307603*	similar to hypothetical protein MGC37914
5	12.45	*Sult2a2*	sulfotransferase family 2 A, dehydroepiandrosterone (DHEA)-preferring, member 2
6	11.8	*LOC100912485*	alcohol sulfotransferase-like
7	11.46	*Eci1*	enoyl-CoA delta isomerase 1
8	11.23	*Prlr*	prolactin receptor
9	9.44	*Otogl*	otogelin-like; INTERACTS WITH valproic acid (ortholog)
10	8.02	*LOC691546*	similar to ubiquitin-conjugating enzyme E2R 2
11	7.38	*Ech1*	enoyl CoA hydratase 1, peroxisomal
12	6.75	*Hamp*	hepcidin antimicrobial peptide
13	6.64	*Rgs16*	regulator of G-protein signaling 16
14	6.6	*Cyp2c12*	cytochrome P450, family 2, subfamily c, polypeptide 12
15	6.45	*Rps27*	ribosomal protein S27
16	6.38	*LOC100910150*	uncharacterized LOC100910150
17	6.29	*Dbp*	D site of albumin promoter (albumin D-box) binding protein
18	6.27	*Cabp2*	calcium binding protein 2
19	6.18	*Rps27l*	ribosomal protein S27-like
20	5.98	*Rps15al4; Rps15a*	ribosomal protein S15a

**Fig. 3:**
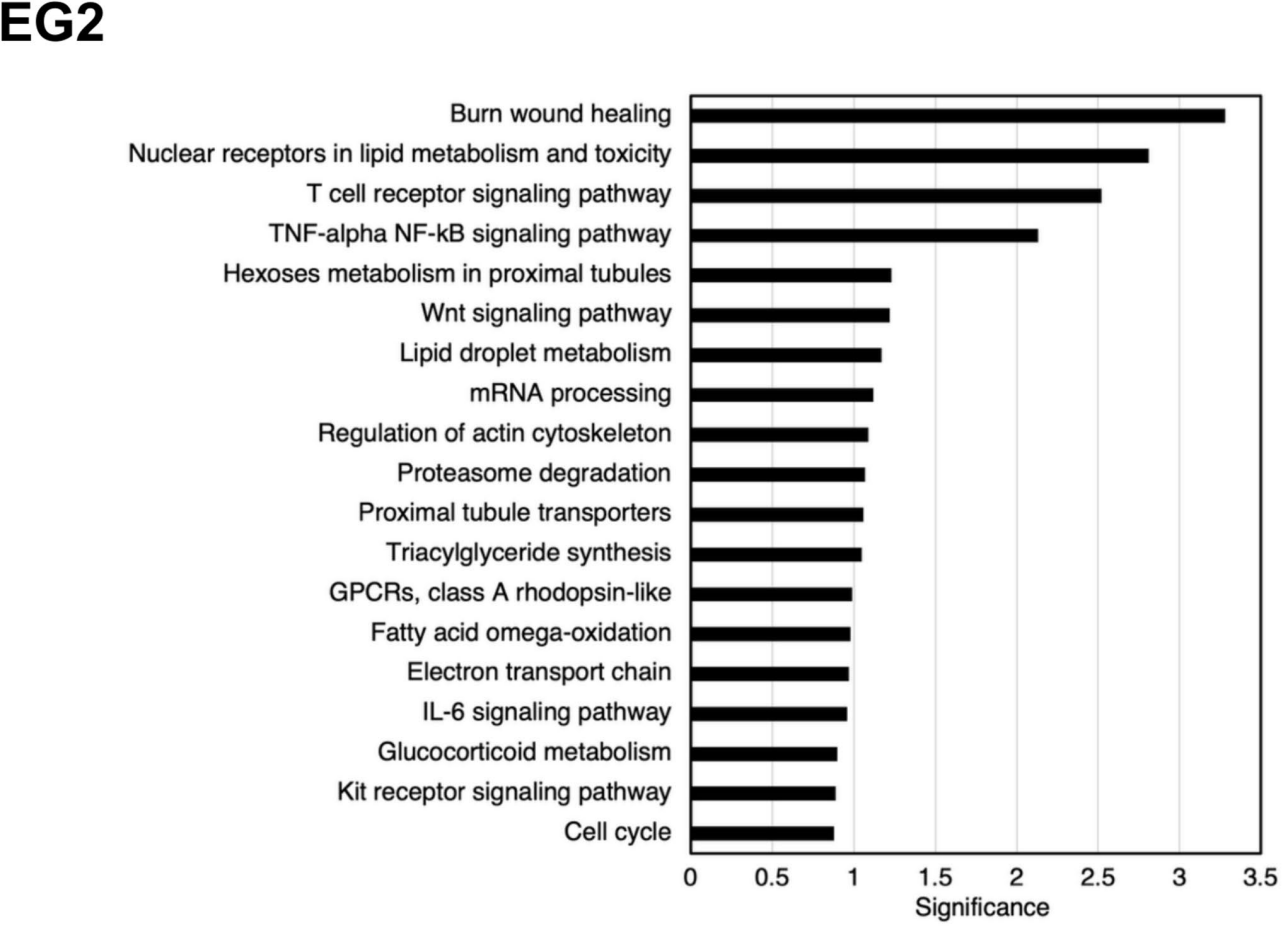
Pathway enrichment analysis (EG2). Pathway enrichment analysis based on DNA microarray results from livers of rats administered EG for 2 days (EG2). The top 20 pathways most affected by EG administration are shown

In the EG2 group, both the most upregulated gene (*SLC3A1*) [27] and the fifth most upregulated gene (*SLC7A13*) [28, 29] were transporters involved in cystine uptake (Table 2). Pathway enrichment analysis showed that the “Burn wound healing” pathway, which includes genes related to apoptosis, inflammation, and collagens, ranked first (Fig. 3).

**Fig. 4:**
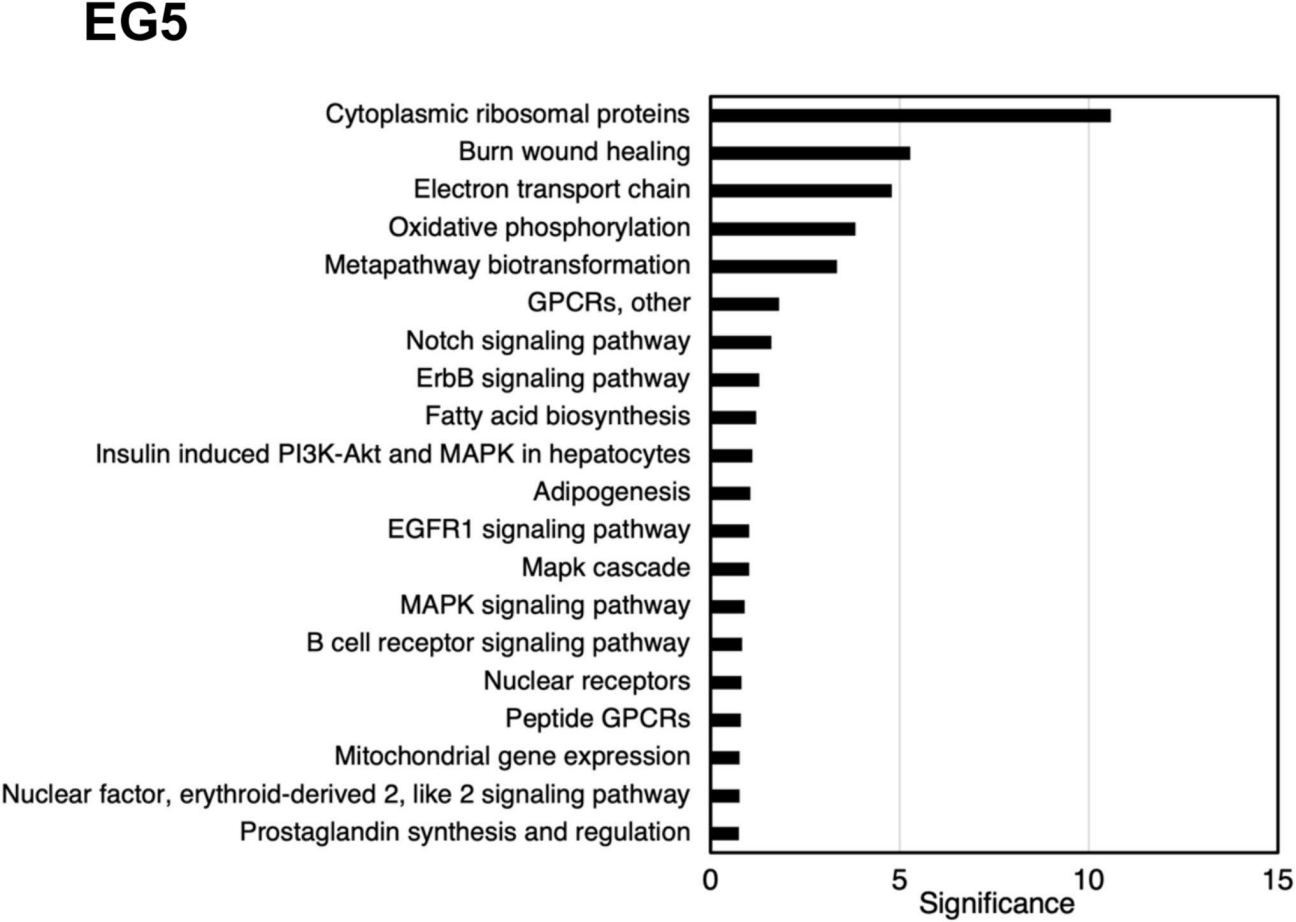
Pathway enrichment analysis (EG5).Pathway enrichment analysis based on DNA microarray results from livers of rats administered EG for 5 days (EG5). The top 20 pathways most affected by EG administration are shown

In the EG5 group, three of the five most upregulated genes (*Sult2a6, Sult2a1,* and *Sult2a2*) were associated with sulfate metabolism and conjugation, which enhance water solubility of xenobiotics for excretion [30, 31], whereas the most upregulated gene (*Akr1b7*) was involved in bile acid generation [32, 33] and the fourth most upregulated gene encoded a glutathione transferase (GST3π) (RGD1307603, Table 3). Pathway enrichment analysis showed that the “Burn wound healing” pathway ranked second in the EG5 groups (Fig. 4). Notably, the “cytoplasmic ribosomal proteins” pathway was ranked first in the EG5 group (Fig. 4). GO enrichment analysis also identified “Ribosome” as the top category in the EG5 group, suggesting increased ribosome synthesis following EG exposure (Supplementary Fig. S5).

Collectively, these findings indicate that cysteine transport may be upregulated as early as 2 days after EG administration. By 5 days, xenobiotic detoxification, which is consisted of chemical modifications of xenobiotics including sulfate conjugation, appeared to be induced, along with increased ribosomal biogenesis, which should be increased during proliferation that may support hepatocyte recovery from EG-induced damage (Figs. 1 and 2).

### Effect of EG metabolism on the SLC7A11-Gpx4 axis in rat liver

**Fig. 5:**
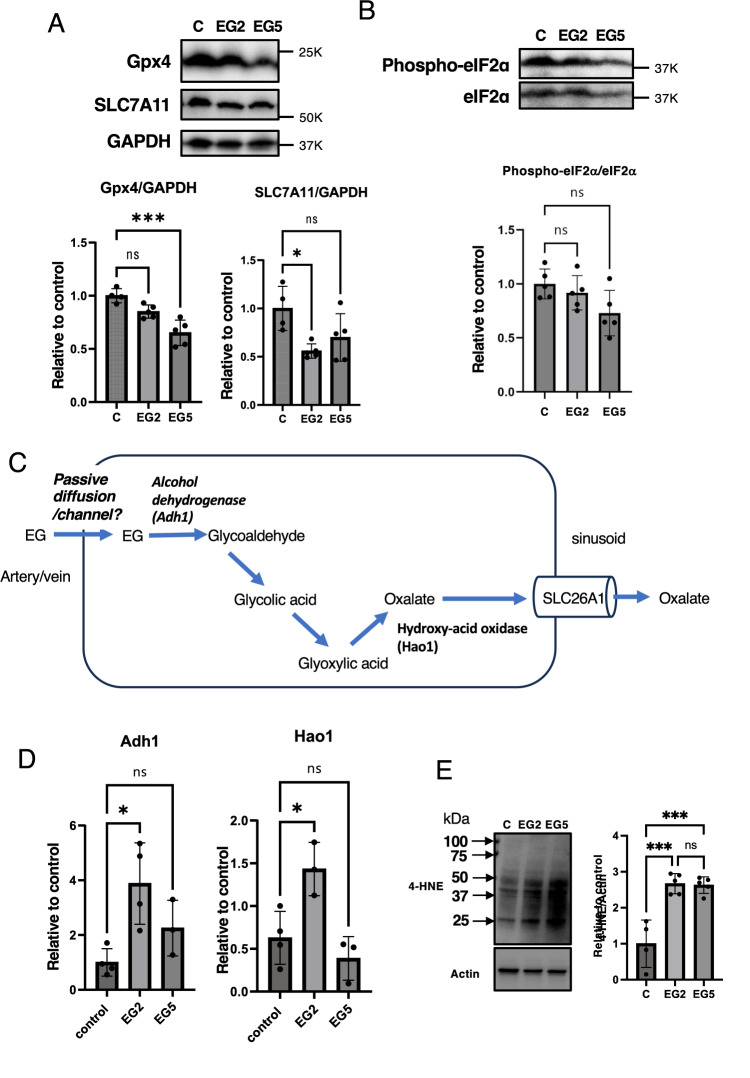
Decreased *SLC7A11*-*Gpx4* axis and increased translational initiation in the liver after EG administration.(**A**, **B**, **E**) Immunoblotting of liver lysates from control rats (**C**) and EG-treated rats 2 (EG2) or 5 (EG5) days after EG administration (8 g/kg) showing levels of the indicated proteins. GAPDH and actin served as loading controls. (**C**) Enzymes and transporter involved in the major steps of EG metabolism and extrusion from hepatocytes. (**D**) Quantitative PCR (qPCR) analysis of Adh1 and Hao1 expression. The graph shows relative levels of indicated mRNAs. Each bar represents the mean ± S.D. ns, not significant; *P < 0.05; ***P < 0.001

The upregulation of *SLC3A1* and *SLC7A13* peaked in the EG2 group and returned to basal levels in the EG5 group (−1.37 for *SLC3A1* and 1.01 for *SLC7A13*). In contrast, another cystine transporter, *SLC7A11*, was downregulated throughout EG administration (−2.07 in EG2 and −3.33 in EG5). Downregulation of this transporter, along with decreased glutathione peroxidase 4 (*Gpx4*), has been shown to result in ferroptosis, a form of regulated necrosis characterized by an iron-dependent increase in lipid peroxidation and reduced cellular capacity to eliminate toxic lipid peroxides [34, 35]. Gpx4 is an only Gpx that can reduce lipid peroxide and SLC7A11 is required for elevation of cellular glutathione levels through providing cysteine, a precursor of glutathione. We thus examined *SLC7A11* and *Gpx4* expression by immunoblotting. As shown in Fig. 5A, *SLC7A11* levels showed a transient but significant decrease followed by partial recovery. In contrast, *Gpx4* levels showed a time-dependent decline, reaching a significant reduction in the EG5 group compared with the control group (Fig. 5A).

We next investigated whether the increased number of nucleoli (Supplementary Fig. S2B) and ribosomal gene expression (Fig. 4 and supplementary Fig. S5) were associated with increased protein synthesis. Immunoblot analysis of eukaryotic initiation factor 2α (eIF2α), whose phosphorylation correlates with inhibition of protein synthesis [36], showed decreased phosphorylation in response to EG administration, indicating increased translation (Fig. 5B). Thus, the increase in nucleolar number might lead to an increase of protein synthesis. To further evaluate the status of hepatic EG metabolism (Fig. 5C), we quantified mRNA levels of enzymes catalyzing major steps of EG metabolism in hepatocytes. As shown in Fig. 5D, both alcohol dehydrogenase (Adh1) and hydroxy acid oxidase (HAO1) were transiently upregulated during EG administration. Given that EG itself is relatively less toxic and its metabolites play major roles in toxicity, these findings are consistent with the transient hepatic damage observed after EG administration (Fig. 1). Finally, we examined 4-hydroxy-2-nonenal (4HNE)-modified protein levels, which reflect lipid peroxidation due to a decrease of SLC7A11-Gpx4 axis [37, 38], and confirmed accumulation of 4HNE-modifed proteins (Fig. 5E), indicative of lipid peroxidation.

### Effect of EG metabolism on the SLC7A11-Gpx4 axis in Huh-7 human hepatoma cells

**Fig. 6:**
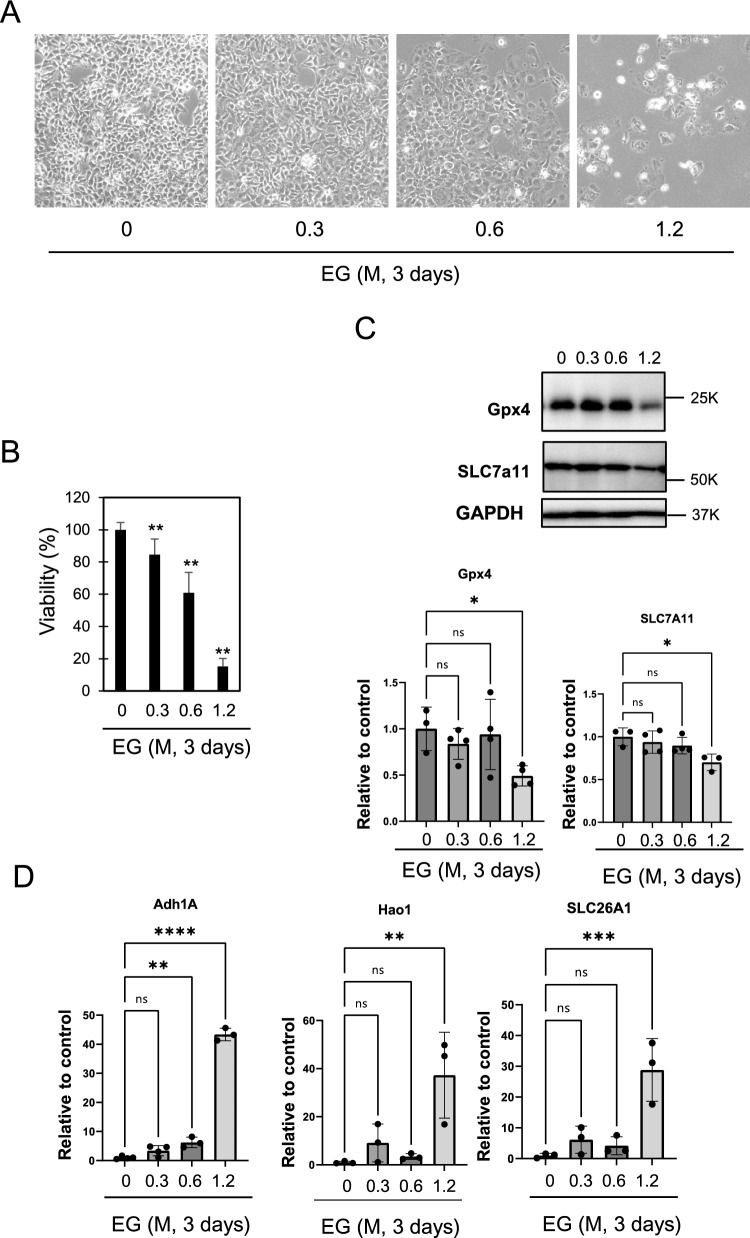
Decreased* SLC7A11*-*Gpx4* axis in Huh-7 human hepatoma cells after EG administration. Morphology (**A**), viability (**B**), immunoblotting (**C**), and qPCR (D) of Huh-7 cells treated with the indicated concentrations of EG for 3 days. M, mole/L. Cell viability was assessed using CCK8 assay. The graph shows relative levels of indicated proteins or mRNAs. Each bar represents the mean ± S.D. ns, not significant; *P < 0.05; **P < 0.01; ***P < 0.001; ****P < 0.0001

To further confirm the negative effect of EG metabolism on the *SLC7A11*-*Gpx4* axis in hepatocytes, EG was administered to Huh-7 human hepatoma cells at concentrations (0.3, 0.6, and 1.2 M) previously reported to produce approximately 50% inhibition of KB cell viability after 3 days [20]. As shown in Fig. 6A and B, cell viability decreased in a concentration-dependent manner. Significant reductions in *SLC7A11* and *Gpx4* levels were also observed in 1.2 M EG group compared with those in the control group, confirming that EG exerts a negative effect on the *SLC7A11*-*Gpx4* axis in hepatic cells (Fig. 6C). Evaluation of EG metabolism, along with solute carrier family 26 member 1 (*SLC26A1*), which is considered the main transporter for oxalate extrusion [39], showed induction of EG metabolism and oxalate extrusion following EG administration (Fig. 6D).

## Discussion

Oral administration of 8 g/kg EG resulted in metabolic acidosis (Table 1), which likely accounted for the cumulative mortality of approximately 25% observed in the experimental group 5 days after administration (2 out of 8). Increased expression of Adh1 and Hao1 was observed in both rat liver and Huh-7 cells following EG administration (Figs. 5 and 6), suggesting an increase in EG metabolites, such as GA and oxalate (Ox), and subsequent metabolic acidosis. Metabolic acidosis may also have contributed to crystal formation in the liver (Supplementary Figs. S3 and S4). However, most animals survived beyond 5 days of exposure (6 out of 8), demonstrating that the liver exhibited substantial resilience against EG toxicity.

The liver is the heaviest organ in the human body, and the liver-to-body weight ratio is strictly maintained compared with other organs [40]. Although liver weights may vary depending on the amounts of glycogen and lipids deposited in hepatocytes, only a 20–25% loss of liver weight has been reported even after 48 h of fasting [41]. Electron microscopic and histological findings revealed glycogen accumulation in hepatocytes of the EG2 group (Fig. 2 and supplementary Fig. S3), suggesting an additional mechanism underlying the decreased liver weights observed in the EG5 group. Considering the increases in AST, ALT, and LDH (Fig. 1), hepatocyte necrosis and subsequent cell loss likely contributed to the decreased liver weights. Serum AST, ALT, and LDH levels peaked in the EG2 group and declined in the EG5 group, whereas loss of liver weight became evident in the EG5 group (Fig. 1 and Table 1). This suggests a delay between hepatocyte necrosis and liver weight loss. Nevertheless, mechanisms other than hepatocyte necrosis may also underlie the observed decrease in liver weight. Since circulating serum ALB is a long-lived protein with a half-life of approximately 2–3 weeks [42], hepatocyte necrosis may not have been reflected as a significant decrease in circulating ALB in our experimental settings (Fig. 2). Alternatively, ALB synthesis may be preserved during EG administration. Notably, liver transplantation in patients with EG poisoning has been repeatedly reported, suggesting that essential liver functions are preserved even after fatal EG poisoning [43–45], in spite of the fact that liver toxicity by EG is also suggest [14, 15].

Giermaziak and Orkisz conducted a detailed analysis of hepatocyte ultrastructure in rats administered EG [15]. They administered EG through a stomach tube at a dose of 5.0 mg/kg to rats and examined ultrastructure of hepatocytes 1, 5, and 14 days after the administration. Although the administration methods used by us and them differs, the doses of EG by both methods correspond to approx. 50% of the lethal dose of EG. Thus, it may be meaningful to compare the two results. They observed irregularly shaped vacuoles predominating in the cytoplasm 1 day after administration [15]. Although they reported large vacuoles with medium electron density in the cytoplasm of sinusoidal endothelial cells 5 days after administration, the main feature of hepatocytes was numerous swollen mitochondria [15]. In our study, although low electron density regions were observed in EG2 hepatocytes, these were not cytoplasmic vacuoles but portions of cytoplasm (Fig. 2, b, e, and h). The low electron density regions contained rosette-like glycogen granules in EG2 hepatocytes, associated with increased fluid content in the cytoplasm, which was reduced in EG5 hepatocytes (Fig. 2, c, f, and i). Because EG is extensively taken up by hepatocytes and metabolized to oxalate, the increased fluid content in the cytoplasm of hepatocytes could consist of EG and its metabolites, including birefringent oxalate crystals (Supplementary Fig. S4, f). One of the most remarkable differences between the observations by us and them is the morphology of mitochondria; they observed swollen mitochondria [15] whilst we did not observe any apparent changes in mitochondrial morphology (Fig. 2). Nevertheless, we do not have any rational explanation about the difference between the observations. Since morphologically normal mitochondria can be sometimes unhealthy, we are planning to examine possible mitochondrial dysfunctions in EG-administered rat kidneys.

Another microscopic finding was the increased occurrence of multiple nucleoli in the nuclei of hepatocytes from EG-administered rat liver (Fig. 2B). As demonstrated in the DNA microarray results, increased ribosomal gene expression was observed in the EG5 samples. Since the nucleolus is the site of ribosomal gene expression, nucleolar multiplication would result in increased expression of ribosomal genes, as demonstrated in this study [46, 47]. Furthermore, we observed a decreased tendency of the Ser51-phosphorylated (inactive) form of eIF2α in hepatocytes from the EG5 group, which may indicate increased translation (Fig. 5B) [48]. Because translation is a highly ATP-consuming process, glycogen accumulated in EG2 hepatocytes might have been depleted 5 days after EG administration, as indicated by PAS and PAM staining (Supplementary Fig. S3, c and f). Several pathways have been suggested to be involved in nucleolar multiplication and subsequent ribosomal biogenesis [49]. Therefore, a key future focus should be to identify the pathway responsible for EG-induced nucleolar multiplication and resultant ribosomal biogenesis, which likely play pivotal roles in hepatic recovery from EG toxicity.

Ferroptosis is an iron-dependent form of necrosis characterized by progressive lipid peroxidation, which ultimately leads to plasma membrane rupture [50, 51]. Although additional mechanisms have been proposed, loss of *Gpx4*-dependent reduction of lipid peroxides, along with glutathione depletion via decreases in *SLC7A11* and/or *Gpx4* (the *SLC7A11*-*Gpx4* axis), was the earliest mechanism identified as a cause of ferroptosis [35, 52]. Ferroptosis has been implicated in the pathogenesis of kidney stone disease, including mechanisms involving CaOx [53, 54]. Oxalate has been repeatedly demonstrated to induce ferroptosis in renal epithelial cells in vitro [55–58]; however, it remains debated whether CaOx directly induces ferroptosis or ferroptosis contributes to CaOx crystal formation and subsequent nephrolithiasis [54]. In the present study, we demonstrated a decrease in the *SLC7A11*-*Gpx4* axis, but we did not establish that this decrease was linked to ferroptosis. However, reduced activity of the *SLC7A11*-*Gpx4* axis likely diminishes cellular antioxidative capacity, which may be harmful to hepatocytes and contribute to the transient hepatic damage observed in this study.

Hyperoxaluria is one of the characteristic features of EG poisoning. During EG intoxication, oxalate is generated in the liver and excreted through an exchange between intracellular oxalate and extracellular sulfate mediated by *SLC26A1* (also known as sulfate anion transporter-1, Sat-1) [59–61]. Thus, it is plausible to speculate that sulfate incorporated into hepatocytes via *SLC26A1* is utilized by sulfotransferases in EG5 hepatocytes. However, the exact role of sulfate conjugation in the elimination of EG and its metabolites remains to be established.

## Conclusions

We demonstrated transient liver damage in an experimental animal model of EG poisoning. Downregulation of the *SLC7A11*-*Gpx4* axis may contribute to EG-induced hepatic injury, while enhanced ribosomal biogenesis might play a role in recovery. These observations might contribute to not only the elucidation of EG toxicity but also forensic investigation of death by poisoning.

## Supplementary Information

Below is the link to the electronic supplementary material.

Supplementary file1

## Data Availability

The authors confirm that the data supporting the findings of this study are available within the article and its supplementary materials.
