# Supplementary material for: Possible role of ribosome biogenesis in the recovery from transient hepatic damage caused by ethylene glycol in rats
Source: Forensic Toxicol. 2026 Feb 3;44(2):313–26. doi: 10.1007/s11419-026-00757-4 (PMC13303457; doi:10.1007/s11419-026-00757-4)
Supplement: Supplementary file 1 — Supplementary file1 [file 11419_2026_757_MOESM1_ESM.docx]

**Supplementary Table S1. Primers used in this study**

| **Primer name** | **Sequence** |
| --- | --- |
| Human-*Adh1A*-forward | CATCCCACTCGCTATTCCTCA |
| Human-*Adh1A* -reverse | GGTCCCCTGAGGATTGCTTAC |
| Human-*Hao1*-forward | GCCAATATGTGTGGGGGCTA |
| Human-*Hao1*-forward | GGCCCAGGAACTCAACATCA |
| Human-*SLC26a1*-forward | GCGGGCGTGACTGCTAC |
| Human-*SLC26a1*-reverse | GGGTGTTGCTGGAAGCTGTTT |
| Human-*GAPDH*-forward | GGTCGGAGTCAACGGATTTGGTCG |
| Human-*GAPDH*-reverse | CCTCCGACGCCTGCTTCACCAC |
| Rat-*Adh1*-forward | ACTGATGGAGGGGTGGACTT |
| Rat-*Adh1*-reverse | GACAATGACGCTTACACCGC |
| Rat-*Hao1*-forward | CATGCCAATATGCGTTGGGG |
| Rat-*Hao1*-forward | CATGCCAGTTCCCATGGTCT |
| Rat-*SLC26a1*-forward | GCTTAGAGGGTGAGGCAACAG |
| Rat-*SLC26a1*-reverse | GACCAGTACCAGTGTCCCG |
| Rat-*GAPDH*-forward | GGTCTCTGCTCCTCCCTGT |
| Rat-*GAPDH*-reverse | TGCCGTTGAACTTGCCGTGGG |

**Supplementary Table S2. Antibodies used in this study**

| **Antibody** | **Supplier** | **Identifier** | **Dilution** |
| --- | --- | --- | --- |
| Anti-SLC7A11 | abcam | ab175186 | 1/1000 |
| Anti-Gpx4 | abcam | ab125066 | 1/1000 |
| Anti-phospho-eIF2α | Cell Signaling Technology | #9721 | 1/1000 |
| Anti-eIF2α | Cell Signaling Technology | #9722 | 1/1000 |
| Anti-GAPDH | Merck | MAB374 | 1/1000 |
| Anti-actin | Sigma | A2066 | 1/1000 |
| Anti-4-hydroxy-2-nonenal | JaICA | MHN-100P | 1/1000 |
